# Regulatory T-Cells Suppress Cytotoxic T Lymphocyte Responses against Microglia

**DOI:** 10.3390/cells11182826

**Published:** 2022-09-09

**Authors:** Priyanka Chauhan, Shuxian Hu, Wen S. Sheng, James R. Lokensgard

**Affiliations:** 1 Neurovirology Laboratory, Department of Medicine, University of Minnesota Medical School, Minneapolis, MN 55455, USA; 2 3-107 Microbiology Research Facility, University of Minnesota, 689 23rd Avenue S.E., Minneapolis, MN 55455, USA

**Keywords:** microglia, regulatory T-cells, cytotoxicity

## Abstract

Regulatory T-cells (Tregs) play pivotal roles during infection, cancer, and autoimmunity. In our previous study, we demonstrated a role for the PD-1:PD-L1 pathway in controlling cytolytic responses of CD8^+^ T lymphocytes against microglial cells presenting viral peptides. In this study, we investigated the role of Tregs in suppressing CD8^+^ T-cell-mediated cytotoxicity against primary microglial cells. Using in vitro cytotoxicity assays and flow cytometry, we demonstrated a role for Tregs in suppressing antigen-specific cytotoxic T-lymphocyte (CTL) responses against microglia loaded with a model peptide (SIINFEKL). We went on to show a significant decrease in the frequency of IFN-γ- and TNF-producing CD8^+^ T-cells when cultured with Tregs. Interestingly, a significant increase in the frequency of granzyme B- and Ki67-producing CTLs was observed. We also observed a significant decrease in the production of interleukin (IL)-6 by microglia. On further investigation, we found that Tregs significantly reduced MHC class 1 (MHC-1) expression on IFN-γ-treated microglial cells. Taken together, these studies demonstrate an immunosuppressive role for Tregs on CTL responses generated against primary microglia. Hence, modulation of Treg cell activity in combination with negative immune checkpoint blockade may stimulate anti-viral T-cell responses to more efficiently clear viral infection from microglial cell reservoirs.

## 1. Introduction

Regulatory T-cells (Tregs), characterized as CD4^+^CD25^+^FoxP3^+^, play important roles in inhibiting effector T-cell function in order to maintain self-tolerance and regulate immunological homeostasis [1,2]. They can be found at elevated densities in many cancers and are thought to be a major barrier to the generation of robust anti-tumor T-cell responses [3]. While Tregs maintain order during autoimmune and inflammatory responses, as well as immune responses generated during viral infection, and are required to minimize tissue damage, imbalance or dysfunction caused by depletion of these cells during viral infection has been demonstrated to lead to pathological tissue damage [4,5,6,7,8,9]. Similar to checkpoint inhibitors on infected target cells, Tregs, when activated at the site of cytotoxic T-lymphocyte (CTL)-mediated killing, have been shown to reduce CTL killing rates, and co-cluster with effector T-cells [10,11]. In our previous study, we demonstrated a role for the PD-1:PD-L1 pathway in controlling cytolytic responses of CD8^+^ T lymphocytes against microglial cells presenting viral peptides. In this study, we investigated whether Tregs contribute to suppression of immune responses against microglial cells.

Microglia are the primary brain-resident sentinel cells of the central nervous system (CNS) [12,13,14]. They appear early during embryogenesis and derive from early myeloid precursors in the embryonic yolk sac [15]. They are the most abundant mononuclear macrophages found within brain parenchyma and subsist for a very long time through slow cell division. It has been reported that the median rate of human microglial cell renewal is around 30% per year [16]. This slow division and long life make microglial cells an ideal viral reservoir within the brain, thus, allowing CNS viral persistence (e.g., HIV) throughout the life of the patient, unlike other shorter-lived, infected cells [12]. Activated microglia also release many cytokines, chemokines, and neurotoxic mediators that may contribute to pathological inflammation [17,18]. It can be said that microglia serve as a perfect brain reservoir for a number of viral infections [12].

We have previously demonstrated that microglial cells govern the activity of brain-infiltrating T-cells through the PD-1:PD-L1 pathway [19]. Similarly, we also found that Tregs limit viral encephalitis through restraining expansion of cytotoxic T-cells within infected brain [20,21]. While immunosuppressive responses mediated by the PD-1:PD-L1 pathway and Tregs within the brain are undoubtedly beneficial to the host, preventing immune-mediated destruction to this vital organ, the establishment of an anti-inflammatory milieu may also result in deficiencies in viral clearance. In our previous publication, we showed that PD-L1 induction on microglia restrains cytotoxic T-lymphocyte responses [22]. In this study, we aimed to investigate the role of Tregs in suppression of these CTL responses against microglial cells. The results generated from these studies will uncover the potential of combined therapeutic blockade, through both inhibiting the PD-1:PD-L1 pathway and modulating Tregs, in promoting CD8^+^ T-cell-mediated clearance of viral infection from glial cell reservoirs.

## 2. Materials and Methods

### 2.1. Ethical Statement

This study was conducted according to the guidelines for Care and Use of Laboratory Animals of the National Institutes of Health. The University of Minnesota’s Institutional Animal Care and Use Committee approved the protocol (Protocol Number: 2001-37741A). All animals were routinely cared for in accordance with RAR (Research Animal Resources) guidelines. Efforts were made to ameliorate animal suffering. Animals were euthanized only after isoflurane inhalation, whenever required.

### 2.2. Experimental Animals and Virus

Pathogen-free OT-1 (JAX stock#003831) and C57BL/6 (JAX stock#000664) mice were purchased from The Jackson Laboratory (Bar Harbor, ME, USA) and housed in individually ventilated cages at RAR, University of Minnesota. Animals were provided with food and water ad libitum. An adenovirus vector expressing ovalbumin (rAd5-OVA) was purchased from Applied Biological Materials Inc. (Richmond, BC, Canada). It is a second-generation vector derived from human adenovirus type 5. OT-1 mice (8–10 weeks old) were primed with rAd5-OVA (1 × 10^10^ PFU/mouse) intravenously to generate cytotoxic T-lymphocytes. C57BL/6 neonatal mice (1-day-old) were used to culture primary microglial cells. C57BL/6 mice (8–10 weeks) were used to isolate Tregs after injecting them with IL-2/anti-IL-2 complex.

### 2.3. Reagents

Recombinant mouse IL-2 and anti-IL-2 mAb (JES6-1A12) were purchased from Biolegend (San Diego, CA, USA). SIINFEKL (i.e., SL8; OVA_257–264_) was purchased from GenScript (Piscataway, NJ, USA).

### 2.4. Injection of Murine IL-2/Anti-IL-2 mAb Complexes

First, 1 µg of murine IL-2 was incubated with 5 µg of anti-mouse IL-2 mAb at 4 °C for 15 min to allow the formation of immune complexes. The complexes of IL-2/anti-mouse IL-2 were then injected into C57BL/6 mice via the i.p route in 100 µL saline for 3 days (1 dose/day) to expand Tregs [23].

### 2.5. Primary Murine Microglial Cell Cultures

Murine cerebral cortexes from 1-day-old mice were dissociated using Trypsin (0.25%) for 30 min and plated in 75 cm^2^ Falcon culture flasks in DMEM containing FBS (5%), penicillin (100 U/mL), streptomycin (100 μg/mL) (Sigma-Aldrich, St. Louis, MO, USA), gentamicin (50 μg/mL), and Fungizone^®^ (250 pg/mL) (Invitrogen, Carlsbad, CA, USA). The medium was replaced after 1 and 4 d of plating. On d 12 of culture, floating microglial cells were collected and used for in vitro CTL assays. Purified microglial cells were >95% positive for Iba-1 and <2% positive for glial fibrillary acidic protein (GFAP) (phenotypic marker of astrocytes) [24].

### 2.6. CFSE-Labeling of Microglial Cells

Microglial cells were labeled with an intravital dye, CFSE (Invitrogen). Briefly, 2 × 10^6^ cells/mL cells were suspended in PBS/5% FBS. Two concentrations of CFSE were made in PBS, CFSE-hi (10 µM) and CFSE-lo (1 µM), which were then added to the cells in a 1:1 ratio [5 µM (hi) or 0.5 µM (lo) working concentrations]. The cells were incubated in a 37 °C water bath for 20 min. The cells were then washed twice with PBS/2% FBS and an aliquot of CFSE-lo-labeled cells was pulsed with 1 µg/mL of SIINFEKL peptide for 30 min at 37 °C based on our previous study [22], and then washed with PBS/2% FBS. This was followed by mixing of CFSE-lo cells (±SIINFEKL) with CFSE-hi-labeled cells in a 1:1 ratio. These CFSE-labeled microglia were used in CTL assays.

### 2.7. Microglial Cell Cytotoxicity Assay

The OT-1 mice were intravenously primed with rAd5-OVA (1 × 10^10^ PFU/mouse) to generate an immune response against ovalbumin. Then, 5 d later, splenocytes were harvested and CD8^+^ T-cells were isolated using the MagCellect Mouse CD8^+^ T-Cell Isolation Kit (R&D Systems, Minneapolis, MN, USA). Purified CD8^+^ T-cells were considered effector cells. During the same time, C57BL/6 mice were injected with IL-2/anti-IL-2 complexes for 3 days and their splenocytes were used to isolate Tregs using a CD4^+^CD25^+^ Regulatory T-cell Isolation Kit (Miltenyi Biotech, Auburn, CA, USA). CFSE-labeled microglia (±SIINFEKL) were used as target cells. Microglia were incubated with Tregs for 1 h and then co-cultured with CD8^+^ T-cells in different Treg:CD8^+^ T-cell ratios (5:1, 2:1, and 1:1) for 18 h. Cells were then trypsinized and used for flow cytometric analysis of the CFSE-hi/CFSE-lo ratios. Specific killing was determined as [1 − (control group ratio/experimental group ratio)] × 100.

### 2.8. Flow Cytometry Analysis

Cells were surface stained using CD8-efluor 450 (53–6.7; 0.25 µg/test; eBioscience, San Diego, CA, USA), CD45-BV605 (30-F11; 0.5 µg/test; Biolegend), CD11b-AF700 (M1/70; 0.25 µg/test; eBioscience), and MHC-1 APC (AF6-88.5.5.3; 0.5 µg/test; eBioscience) prior to permeabilization and fixation using the Cytofix/cytoperm kit (eBioscience). Cells were then stained for IFN-γ-BV650 (XMG1.2; 0.25 µg/test; Biolegend), TNF-BV711 (MP6-XT22; 0.5 µg/test; Biolegend), Granzyme B-AF647 (GB11; 5µL/test; Biolegend), Ki67-PE-Cy7 (SolA15; 0.125 µg/test; eBioscience), and IL-6-PerCP-eFluor710 (MP5-20F3; 0.125 µg/test; eBioscience) according to the manufacturer’s protocol.

### 2.9. Statistical Analyses

GraphPad Prism software was employed to determine statistical significance (version 9.3; Graphpad Software, La Jolla, CA, USA). For comparing groups, an ordinary one-way ANOVA with multiple comparisons and Student’s two-tailed unpaired *t*-test were used. A *p* value < 0.05 was considered significant.

## 3. Results

### 3.1. IL-2/Anti-IL-2 Complex Efficiently Expanded Tregs

**Figure A1:**
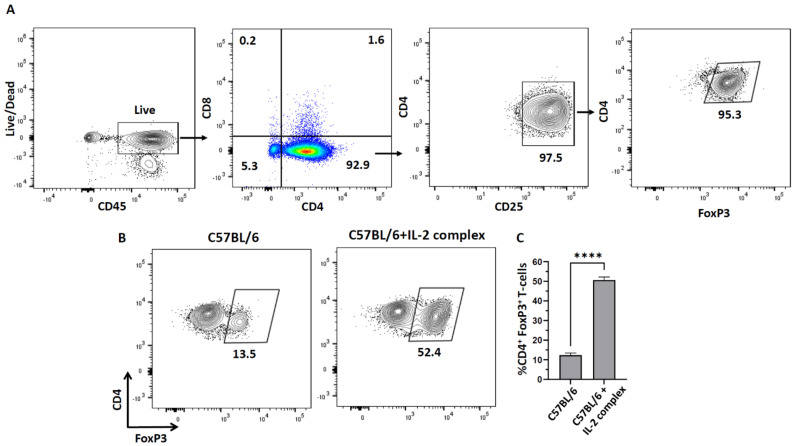
**IL−2/anti−IL−2 complex expands the Treg population**. Mice were either injected with IL−2/anti−IL−2 complex or left as control for 3 consecutive days after which splenocytes were isolated and analyzed for Tregs using flow cytometry. (**A**) Gating strategy. Tregs were isolated from the spleen of C57BL/6 mice injected with the IL−2/anti−IL−2 complex. The cells were then analyzed for live/dead, CD4, CD8, CD25, and Foxp3 expression. Freshly isolated Tregs were assessed for their purity before the experiments were carried out. (**B**) Representative contour plots of CD4^+^CD25^+^ Foxp3^+^ Tregs after injecting animals with the IL−2/anti−IL−2 complex. (**C**) Bar graph showing the % of CD4^+^CD25^+^Foxp3^+^ T-cells after injecting the IL−2/anti−IL−2 complex. The experiment was performed 3 times with *n* = 3 and the data are presented as mean ± SE. **** *p*  <  0.0001.

To validate that IL-2/anti-IL-2 complex can significantly expand the Treg population in vivo, we administered IL-2/anti-IL2 complexes to C57BL/6 mice for 3 days and harvested splenocytes for Treg isolation. As shown in the Figure A1, administration of the IL-2/anti-IL-2 complex significantly increased the percentage of CD4^+^Foxp3^+^ Tregs (50.7 ± 0.8%) compared to the control animals (12.4 ± 0.8%; Figure A1B,C). The purity of freshly isolated Tregs was also verified by staining with anti-CD4 and anti-CD25 antibodies and flow cytometry. As shown in the Figure A1, the purity of isolated Tregs was greater than 95% and the majority of cells expressed Foxp3, the signature marker of Tregs (Figure A1A).

### 3.2. Role of Tregs in Suppressing CTL Activity against Microglia

**Figure 1:**
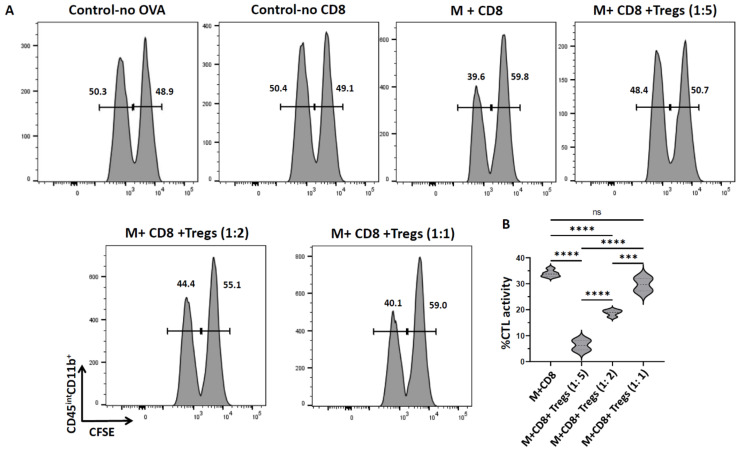
**Tregs suppress cytotoxic T-lymphocyte activity against primary microglia**. CD8^+^ T−cells were isolated from the spleens of rAd5−OVA−primed OT−1 mice using a negative selection kit. CD8^+^ T−cells were then co−cultured with microglial cells (10:1) loaded with ovalbumin−specific SIINFEKL peptide and labeled with CFSE (M). Microglia were pre−incubated with Tregs for 1 h in different ratios to CD8^+^ (CD8^+^:Tregs: 1:5, 1:2, and 1:1). In vitro cytotoxicity was determined after 18 hr. (**A**) Representative histograms show CFSE-labeled microglia harvested from the plate after 18 hr of co-culture with OT−1 CD8^+^ T-cells ± Tregs. Control is CFSE staining of microglia either without a specific peptide or without CD8^+^ T−cells. (**B**) Graphical representation of % CTL activity [1 − (control group hi/lo ratio: experimental group hi/lo ratio)] × 100. *** *p*  <  0.001, **** *p*  <  0.0001. Data shown are representative of three separate experiments using 3 wells/group/experiment and expressed as mean  ±  SE. rAd-OVA-primed OT-1 mice (*n* = 2/experiment) and IL−2/anti−IL−2−injected C57BL/6 mice (*n* = 4/experiment).

To investigate the role of Tregs in controlling CTL activity against microglia, we employed an in vitro CTL assay using a model antigen, ovalbumin (OVA), as described in our previous publication [22]. In these studies, primary murine microglia were first CFSE-labeled and then pulsed with the SIINFEKL (OVA_257–264_) peptide. CD8^+^ T-cells were obtained from the OT-1 T-cell receptor transgenic mice, which had been primed 5 d previously with rAd5-OVA. Tregs were isolated from C57BL/6 mice which were injected with the IL-2/anti-IL-2 complex for 3 days as described in the previous section. Firstly, Tregs were pre-incubated with SIINFEKL-pulsed primary microglia (target cells) for 1 h and then OVA-specific CD8^+^ T-cells (effector cells) were mixed with the microglia/Tregs at CD8^+^T-cells to Tregs ratios of 1:5, 1:2, and 1:1. The effector (CD8^+^ T-cells) to target (microglia) ratio was 10:1 as determined in our previous publication [19]. Co-cultures were incubated for 18 h and CTL activity was assessed using flow cytometry. When SIINFEKL-pulsed microglia were incubated with CD8^+^ T-cells, we observed 34.0 ±0.6% CTL activity. This CTL activity was significantly reduced when microglia were pre-incubated with Tregs in 1:5 (6.3 ± 1.7%) as well 1:2 (18.8 ± 0.6%) ratios of CD8^+^ T-cells:Tregs. However, we did not observe any suppression of CTL activity when Tregs were added at the same ratio as CD8^+^ T-cells (1: 1), (Figure 1).

### 3.3. Tregs Modulate Pro-Inflammatory Cytokine Production

**Figure 2:**
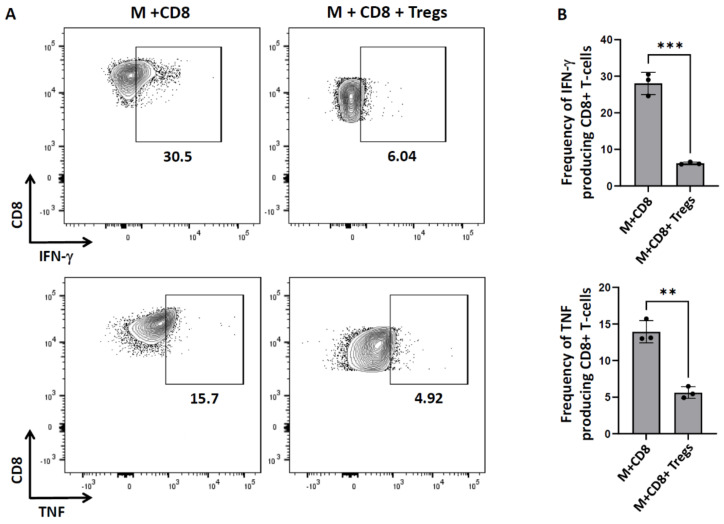
**Tregs modulate pro−inflammatory cytokines.** In vitro CTL assays were carried out as described previously in Figure 1 using a 1:5 ratio of CD8^+^:Tregs. After 18 h of co-culture, CD8^+^ T−cells were analyzed using flow cytometry. (**A**) Representative contour plots show production of the pro−inflammatory cytokines IFN-γ as well as TNF. (**B**) Bar graphs show the frequency of IFN-γ− and TNF−producing CD8^+^ T−cells. Data shown are representative of three experiments using 3 wells/group/experiment and expressed as mean  ±  SE. rAd-OVA-primed OT-1 mice (*n* = 2/experiment) and IL−2/anti−IL−2−injected C57BL/6 mice (*n* = 4/experiment). ** *p*  <  0.01, *** *p*  <  0.001.

We investigated the production of the pro-inflammatory cytokines IFN-γ and TNF by cytotoxic T-lymphocytes when co-cultured with microglia in the presence of Tregs using the same CTL assay described above. We observed that 28.0 ± 1.7% of CD8^+^ T-cells secreted IFN-γ when cultured with microglia. However, this IFN-γ production was significantly reduced in the presence of Tregs (6.2 ± 0.4%). Similarly, we observed a significant reduction in the TNF production in the presence of Tregs (5.6 ± 0.5%), as compared to the control (14.0 ± 0.8%; Figure 2).

### 3.4. Tregs Augment Granzyme B Production and T-Lymphocytes Proliferation

**Figure 3:**
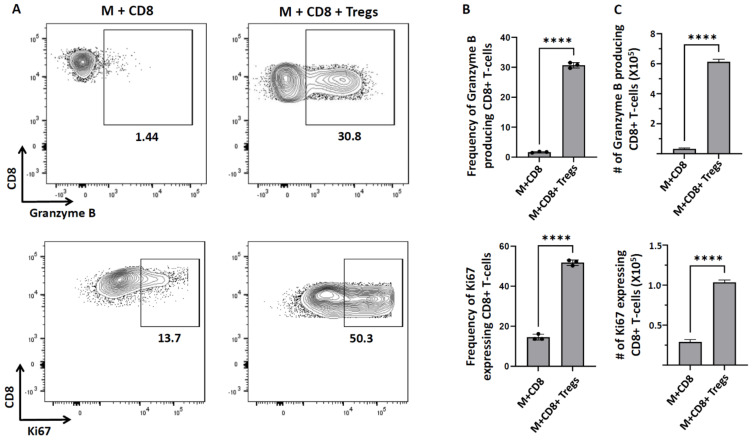
**Tregs augment granzyme B production and T−lymphocyte proliferation.** CD8^+^ T−cells were analyzed for the production of granzyme B and Ki67 after 18 h of co-culture with microglia in the presence or absence of Tregs. (**A**) Representative contour plots show expression of granzyme B and Ki67. (**B**) Bar graphs show the frequency of granzyme B− and Ki67−producing CD8^+^ T−cells. (**C**) Bar graphs show the number of granzyme B− and Ki67−producing CD8^+^ T−cells. Data shown are representative of three experiments using 3 wells/group/experiment and expressed as mean  ±  SE. rAd−OVA−primed OT−1 mice (*n* = 2/experiment) and IL−2/anti−IL−2-injected C57BL/6 mice (*n* = 4/experiment). **** *p*  <  0.0001.

We next investigated the production of granzyme B by CD8^+^ T-cells when co-cultured with SIINFEKL-loaded microglia in the presence of Tregs. Interestingly, we observed an increased proportion of cells producing granzyme B (30.6 ± 0.5%) in the presence of Tregs as compared to the control (1.6 ± 0.1%). Further, we analyzed CD8^+^ T-cell proliferation by staining with Ki67. We observed that CD8^+^ T-cells exhibited significantly enhanced proliferation when co-cultured with microglia and Tregs (51.8 ± 0.7%) as compared to microglia alone (14.5 ± 0.8%; Figure 3).

### 3.5. Tregs Modulate Microglial Secretion of IL-6

**Figure 4:**
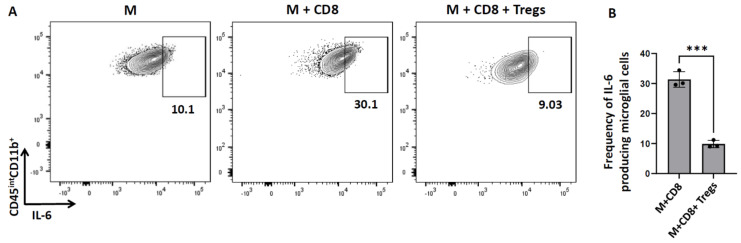
**Tregs modulate microglial cell production of IL−6**. Microglial cells were trypsinized from the in vitro cytotoxicity assay plates and further analyzed for IL−6. (**A**) Representative contour plots show production of IL−6 by microglia (CD45^int^CD11b^+^). (**B**) Bar graph showing the frequency of IL−6−producing microglial cells. Data shown are representative of three experiments using 3 wells/group/experiment and expressed as mean  ±  SE. rAd-OVA-primed OT-1 mice (*n* = 2/experiment) and IL−2/anti−IL−2-injected C57BL/6 mice (*n* = 4/experiment). *** *p*  < 0.001.

Furthermore, we investigated the effect of Tregs on microglial cell production of pro-inflammatory cytokines such as IL-6. We observed that 31.6 ± 1.5% of activated microglia produced IL-6 in the presence of CD8^+^ T-cells. However, in the presence of Tregs there was a significant reduction in IL-6. We demonstrated that 9.8 ± 0.6% of microglia produced IL-6 when co-cultured with Tregs and CD8^+^ T-cells (Figure 4).

### 3.6. Tregs Reduce Class 1 MHC Expression on Microglia

**Figure 5:**
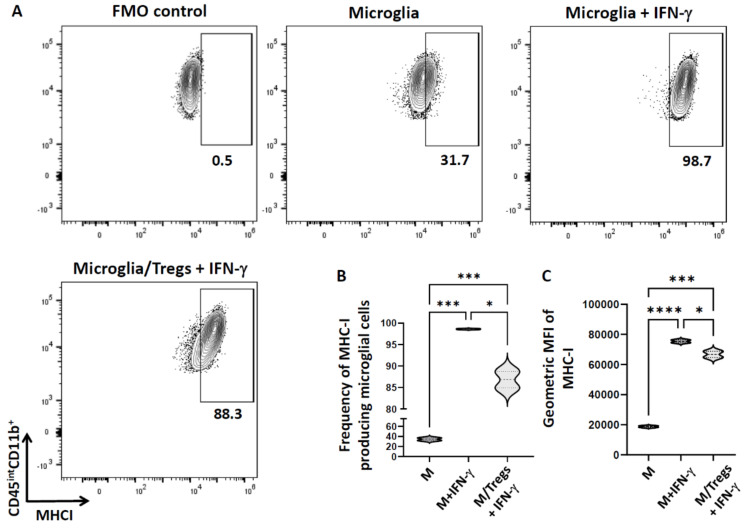
**Tregs reduce MHC class I expression on microglial cells.** Microglia were incubated with Tregs for 1 h and then co-cultured with IFN-γ (10 ng/mL) overnight, after which cells were harvested and analyzed for the expression of MHC−1. (**A**) Representative contour plots show expression of MHC−1 by microglia (CD45^int^CD11b^+^). (**B**) Violin plot showing the frequency of MHC−1-producing microglial cells. (**C**) Violin plot showing the geometric MFI of MHC−1. Data shown are representative of three experiments using 3 wells/group/experiment and expressed as mean  ±  SE. IL−2/anti−IL−2-injected C57BL/6 mice (*n* = 4/experiment). **** *p*  <  0.0001, *** *p*  < 0.001, and * *p*  < 0.05.

Finally, we analyzed expression of class 1 MHC molecules (MHC-1) on microglia when cultured with Tregs. We observed that 34.3 ± 2.6% of primary microglia constitutively expressed MHC-1. When these cells were treated with IFN-γ, almost all the treated microglia expressed MHC-1 (98.6 ± 0.1%). However, when these activated microglia were cultured with Tregs, a reduction in the expression of MHC-1 (86.8 ± 1.9%) was observed, which was statistically significant (Figure 5).

## 4. Discussion

One of the highest priorities in modern virus research is to identify strategies in which viral load is fully suppressed for an extended period in the absence of antiviral therapy. In our previous publication, we have shown that infected microglia are viable cellular targets for immunotherapy [22]. We reported how the PD-1:PD-L1 negative immune checkpoint pathway interferes with complete clearance of virus from microglial cell reservoirs [22]. In addition to checkpoint blockade, Tregs expressing the transcription factor Foxp3 are an attractive target for immunotherapy [25]. Tregs are well-known to a play crucial role in suppression of immune responses during infection and cancer, as well as autoimmunity, and several studies describe their role in anti-viral immunity [26,27]. A number of previous studies have reported that the presence of Tregs within the brain has a significant impact on neuroinflammation, where they generally serve to limit tissue damage [28]. It has also been shown that depletion of the Treg population leads to more robust generation of effector T-cells in response to viral infection [29,30]. Moreover, we have previously demonstrated that accumulation and retention of immunosuppressive Tregs results in concomitant neuroinflammation [20,31]. In this study, we assessed the role of Tregs on CTL against glial cell reservoirs using a model antigen.

The low number of Tregs that can successfully be isolated from naïve animals restricts their application for experimental use. Hence, in this study, we successfully expanded the Treg population using IL-2/anti-IL-2 complexes (Figure A1B,C) and determined their purity following isolation to be over 95% (Figure A1A). This observation is in concordance with other previous findings where the IL-2/IL-2 antibody complex was used to expand Tregs against transient ischemic stroke [23]. It has also been reported that IL-2/anti-IL-2 treatment not only expands the number of Tregs, but also enhances their functions. Hence, we went on to employ these purified Tregs from C57BL/6 mice injected with an IL-2/anti IL-2 complex to study their role in suppressing CTL activity against microglia using a model antigen system. When specific peptide (SIINFEKL)-loaded primary microglia were cultured with CD8^+^ T-cells from OT-1 T-cell receptor transgenic mice, we observed significant cytolytic activity similar to that observed in our previous report [22]. However, in the presence of Tregs, there was a significant reduction in this CTL activity (more than 80% reduction when the Tregs:CD8^+^ ratio was 5:1). Moreover, this immunosuppression was found to be dose-dependent on the ratio of Tregs:CD8^+^. (Figure 1). It has previously been reported that Tregs inhibit antigen-specific CTL killing of a target cell monolayer using a luminescence-based cytotoxicity assay. However, that study employed NIT-1 (an insulinoma cell line established from the islet beta cells) and 8.3 T-cells (CD8^+^ T-cells that recognize the IGRP_206–214_ epitope derived from the islet antigen IGRP (islet-specific glucose-6-phosphatase catalytic subunit-related protein)) as target and effector cells, respectively [32]. In our study, we used primary murine microglial cells loaded with specific peptide (SIINFEKL) as target cells and ovalbumin-specific, TCR transgenic CD8^+^ T-cells as effectors. Although twice the number of Tregs as compared to CD8^+^ T-cells was needed to demonstrate their inhibitory effect, these studies demonstrate that Tregs play a role in controlling CTL responses against microglia. These data provide evidence that modulation of Tregs may be used to synergize with immune checkpoint blockade to stimulate responses against microglial cells (e.g., in immunotherapeutic clearance of HIV-1 brain reservoirs).

We also analyzed the CTLs for their production of the inflammatory cytokines IFN-γ and TNF. Cytotoxic T-lymphocytes produce pro-inflammatory cytokines to help mediate anti-viral responses [33]. However, when we co-cultured these CD8^+^ T-cells with Tregs, we observed a significant drop in their IFN-γ and TNF production (Figure 2). Thus, Tregs exert their immunosuppressive function by inhibiting T-cell activation, cytotoxicity, and secretion of pro-inflammatory cytokines. There are previous studies which demonstrate that T-cells stimulated in the presence of TGF-β (which leads to the induction of Foxp3), result in a suppressive phenotype [2,34]. There are additional published studies which also demonstrate the ability of Tregs to regulate effector T-cell function [35,36].

It has been reported that Tregs regulate immune responses through production of granzyme B and perforins which induce apoptosis of effector cells, thus, decreasing effector number [36]. In the present study, we observed increased granzyme B within the CD8^+^ T-cells, in response to peptide-loaded microglia, when Tregs were present (Figure 3). At this time, we do not understand this finding, as it is contrary to our hypothesis. However, it is possible that the elevated levels observed may reflect granzyme B which is retained within CTLs in the presence of Tregs (i.e., associated with decreased cytotoxic activity). Likewise, the increased CD8^+^ T-cell proliferation may be a feedback mechanism employed by the effector cells to maintain some level of target cell killing in the presence of Tregs. It is also possible that secretion of granzyme B and Ki67 production are time dependent.

Activated microglia not only express the IL-6 receptor, but also potently secrete IL-6 [37,38]. It is well-established that these cells produce exaggerated amounts of pro-inflammatory cytokines, including IL-6, resulting in extreme sickness behaviors, and that Tregs possess the ability to suppress such behavior [37]. In this study, we observed that IL-6 production by microglia in the presence of cytotoxic T-lymphocytes is significantly reduced in the presence of Tregs (Figure 4). Hence, Tregs modulate microglial cell production of IL-6. It is known that antigen presentation on MHC molecules is essential for the generation of adaptive immune responses. Moreover, many cancers evade immune control by losing MHC 1 antigen presentation, thereby impairing the ability of natural immune responses [39]. In our study, we observed that Tregs significantly reduced MHC-1 expression on IFN-γ-treated microglia (Figure 5). This finding suggests that Tregs can also act directly on microglial cells to alter MHC-1 levels, and therefore, their peptide loading capacity, thus, affecting antigen-specific killing by CTLs. We also observed that the fluorescent intensity of CD8^+^T-cells decreased in the presence of Tregs. It has been reported that after activation, CD8+ T-cells form large clusters, however, in the presence of Tregs, cluster formation completely disappeared [32]. We made a similar microscopic observation. It is possible that clustering may, at least in part, explain the differences in intensities.

Taken together, we demonstrated the capacity of Tregs in controlling CTL responses against primary microglial cells. This mechanism likely protects critical brain cells from overzealous cytotoxic immunity but can also be exploited by persisting viruses. In a murine glioma model, synergistic curative activity was observed with Treg modulation along with immune checkpoint blockade [40]. Hence, modulation of Treg cell activity in combination with negative immune checkpoint blockade may further stimulate anti-viral T-cell responses against microglial cell reservoirs to more efficiently clear viral brain infection.

## Data Availability

Data is contained within the article.
